# Surgical Management of an Osteomyelitis Associated Subchondral Bone Defect in the Pediatric Knee Based on Arthroscopy, “Ossoscopy” and Bone Grafting—A Case Report

**DOI:** 10.3390/life12111754

**Published:** 2022-11-01

**Authors:** Christian D. Weber, Filippo Migliorini, Heide Delbrück, Frank Hildebrand

**Affiliations:** Department of Orthopedic Surgery, Trauma and Reconstructive Surgery, RWTH Aachen University Medical Center, Pauwelsstr. 30, 52074 Aachen, Germany

**Keywords:** pediatric knee, repetitive trauma, subchondral defect, septic arthritis, epiphyseal osteomyelitis

## Abstract

Subchondral bone defects around the knee joint are uncommon in skeletally immature patients. These lesions require comprehensive management, especially if related to periarticular bacterial infections. While pediatric osteomyelitis typically affects the metaphysis of long bones, the epiphysis is also a potential site for pyogenic osteomyelitis. Long-term sequelae may include growth plate injury and articular cartilage degradation. Primary epiphyseal subacute osteomyelitis is an extremely rare condition, mainly affecting neonates or young infants, as the cartilage of the growth plate generally acts as a barrier for pathogens. Radiographically, the lesions may appear radiolucent or lytic and often demonstrate a substantial perilesional bone marrow edema in MRI studies, but do not primarily contact the articular surface. However, if diagnosis and treatment of epiphyseal infections are delayed or missed, abscess formation may spread into the knee joint and progress to septic arthritis. Approaching a distal femoral epiphyseal lesion or subsequent bone defect surgically may be limited anatomically by both the subchondral plate and articular cartilage on the distal side and the growth plate proximally. Of the few reported cases of epiphyseal osteomyelitis, most underwent non-operative treatment including antibiotic coverage, or (staged) aggressive surgical care involving open curettage, irrigation and bone grafting. We report a novel combination of arthroscopic techniques, namely “ossoscopy”, bone grafting and antibiotics, to approach a large lateral femoral epiphyseal lesion with knee involvement. In this case report, we present a 5-year old male patient with subacute posttraumatic knee pain and a significant bone defect of the lateral femoral epiphysis related to pyogenic osteomyelitis. The knee joint and periarticular bone lesion were both debrided and irrigated based on arthroscopic and ossoscopic techniques.The osseous lesion was filled with bone graft. The single-stage procedure proved to be a viable treatment to restore both the large subchondral bone defect and full knee function. Over a course of two years, no recurrent symptoms, infection or growth disturbances were observed in the individual.

## 1. Introduction

Subacute osteomyelitis of long bones represents a significant problem among the pediatric population and may infrequently be associated with a radiolucent epiphyseal or subchondral defect [1]. In the United States, an increasing frequency has been reported for children with combined osteomyelitis and septic arthritis (0.8 to 1.3 per 100,000) [2]. Previous studies reported variable rates for associated joint infections in primary epiphyseal osteomyelitis, ranging from 17.7 to 30% [3,4], depending on the pathogen and time interval until treatment was initiated. Epidemiologic studies have identified *Staphylococcus aureus* as the most common cultured pathogen and observed twice as many boys as girls in cases of acute hematogenous osteomyelitis. A history of trauma has been reported in 30% of affected children [4,5]. The bacterial etiology appears to be age-dependent in primary subacute hematogenous osteomyelitis, as the infantile form (6 months–4 years) is often related to *Kingella kingae* and the juvenile form (>4 years) mainly involves *Staphylococcus aureus* [6,7]. Moreover, geographic considerations might play an important role in the observed epidemiology, the type of pathogen identified [1,8,9,10] and the type of detection method applied (e.g., culture vs. organism-specific polymerase chain reaction (PCR) assay) [11]. Pediatric patients with infectious lesions near growth centers should undergo long-term monitoring to ensure the absence of growth disturbances [12]. This should be especially valid for problematic pathogens [12]; Yoo et al. reported a series of eight children with primary epiphyseal osteomyelitis caused by mycobacterium species and observed an intra- and extra-articular spread in five cases, physeal damage in four children and one child with femoral shortening and valgus angulation after a follow-up of 94 months [10]. Post-inflammatory limb deformities and shortening may require multifocal and staged surgical procedures for limb lengthening or deformity correction. 

We present a case of subacute epiphyseal pyogenic osteomyelitis of the lateral femoral condyle with penetration into the knee joint. To the best of our knowledge, the novel treatment combination of empiric first-line iv treatment, knee arthroscopy, “ossoscopy” of the lateral femoral epiphysis via a pre-existing orifice for debridement, sampling, irrigation and bone grafting, has not been reported to date. When compared to previously applied and reported techniques, accessory bone tunnels are not required within the surgery and the use of radiographic (e.g., computed tomography) guidance was omitted. Therefore, both the surgical burden and radiation exposure could be significantly reduced by this minimally invasive single-staged approach. 

## 2. Case Presentation

**Figure 1:**
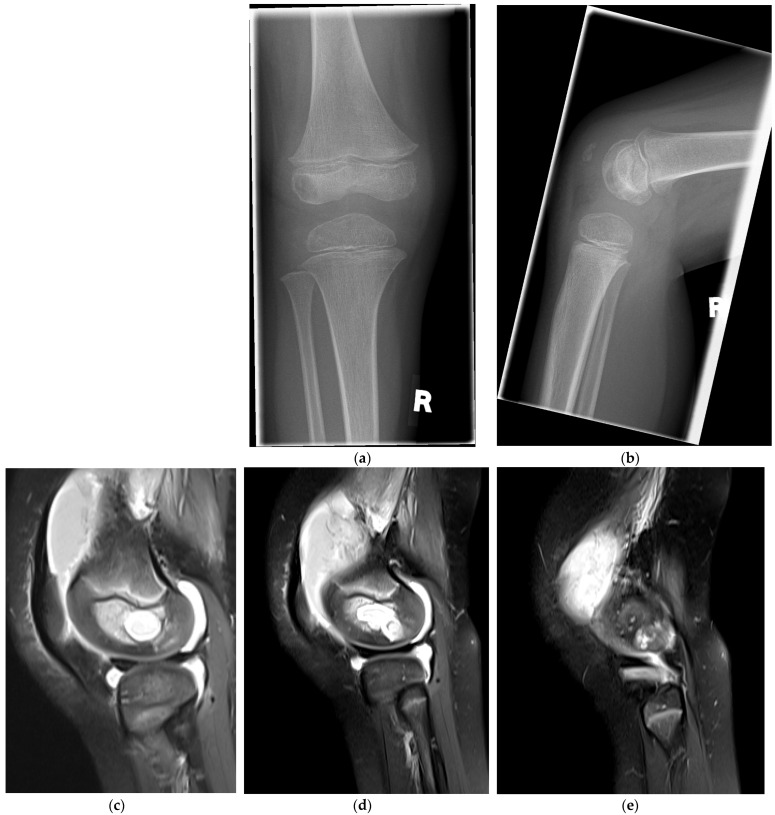
Plain radiographs of the right knee in anterior-posterior (**a**) and lateral (**b**) views after hospital admission, indicating the bone defect in the lateral femoral epiphysis reaching from the growth plate to the subchondral plate. Sagittal MRI (turbo-spin echo, fat saturation) demonstrating a centrally round bone defect with surrounding edema (**c**), in the lateral epiphysis, the defect reaching the subchondral bone (**d**) and penetrating into the knee through a cortical orifice (**e**).

A five-year old boy was transferred to our musculoskeletal referral center from the pediatric department of a local hospital with persistent pain and effusion of the right knee. Three months prior, he had sustained a fall on the right knee from a trampoline. Two months after the injury, the child started to complain again of a painful right knee. He presented with an intermittent limp and finally was unable to bear weight at all and the knee was warm and swollen. During primary clinical evaluation at the pediatric clinic, flexion and extension of the knee were painful and limited. The arthrosonography of the knee revealed an effusion and free fibrin structures within. Plain radiographs showed significant bone loss in the lateral femoral epiphysis reaching from the growth plate to the subchondral plate (Figure 1a,b). The past medial history included an attention-deficit/hyperactivity disorder (ADHD); for this reason, the MRI was obtained under sedation. The distal femoral epiphysis showed a large radiolucent defect with fine sclerotic margins (Type V), according to the definition by Roberts et al. [13], surrounding bone marrow edema and a knee joint effusion (Figure 1c,d). A connection through the lateral cortical wall into the knee was suspected (Figure 1e). The contrast enhancement and edema were indicative of an osteo-myelitic abscess. Single reactive lymph nodes were identified in the dorsal distal thigh. 

In the laboratory workup, both c-reactive protein (CRP 47 mg/L, normal range 0–5) and erythrocyte sedimentation rate (ESR 28, normal range 0–15) were elevated. Initial medical therapy was established with ampicillin/sulbactam 3 × 1 g after suspicious joint aspiration and two days later clindamycin 3 × 250 mg, after obtaining the MRI suggestive for osteomyelitis. Multiple serologic analyses were evaluated as negative (e.g., Borrelia, Mycoplasma, Chlamydia, Yersinia); in addition, the following values were determined: rheumatoid factor 15 IU/mL, ASL < 25, ANA < 1:100, C3 complement 255 mg/dL, C4 complement 45 mg/dL. The joint fluid with turbid appearance was aspirated and synovial white blood cell count was 39.000/µL, with 97% granulocytes, 3% lymphocytes, pH 7.5. Blood cultures remained negative. Distal femoral epiphyseal osteomyelitis with septic arthritis were suspected and after 3 days of pediatric in-hospital workup and care the patient was transferred to our center for surgery. 

**Figure 2:**
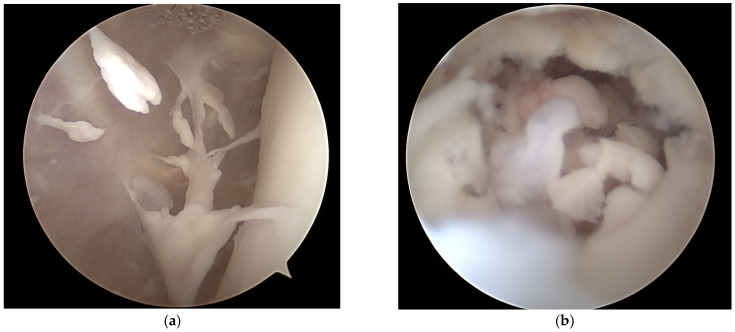

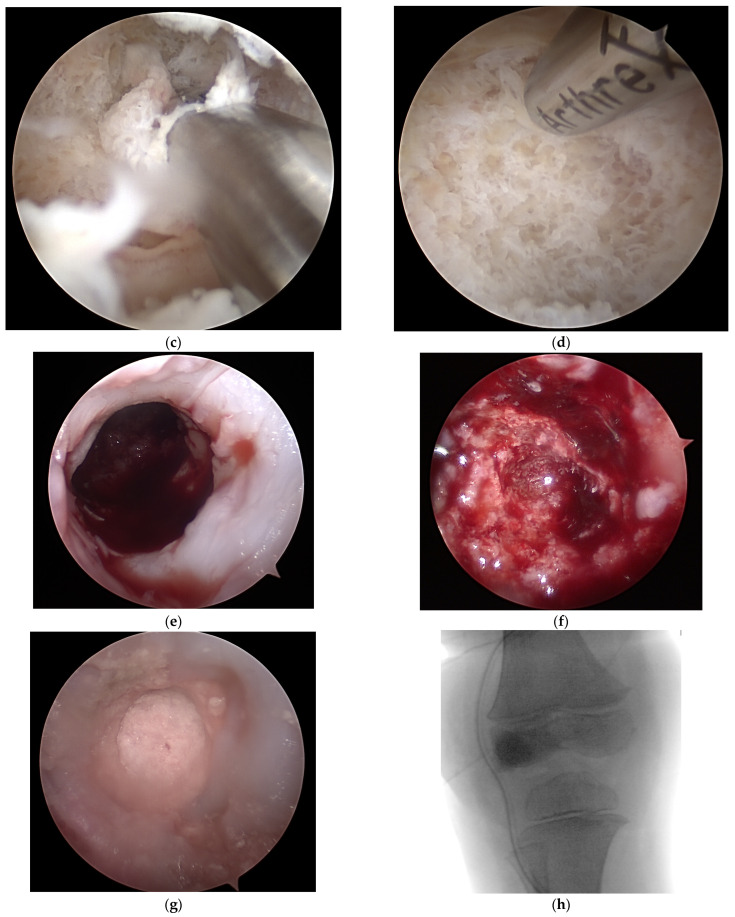
Synovitis and fibrinous deposition were observed in the lateral gutter of the knee joint (**a**), debris in the defect was identified (**b**) and removed with an arthroscopic shaver (**c**), until healthy tissue remained (**d**). The opening of the lesion to the lateral gutter (**e**) and the cavity surrounded by vital bone (**f**), filled by synthetic bone graft (**g**) and complete void filling, was also documented radiographically (**h**).

The day after admission the procedure was scheduled. Under general anesthesia, the patient underwent arthroscopic evaluation of the entire knee joint. The cartilage proved to be intact. Hypertrophic synovitis and fibrin structures were observed especially in the lateral gutter, representing stage II according to Gächter (Figure 2a). The suspect tissues were debrided and samples were obtained. The orifice to the bone defect was then localized in the posterior lateral gutter and after introducing a cannula for optimal trajectories two close-by accessory portals were established, while protecting the medial collateral ligament complex. The 30° arthroscope and a shaver were then introduced into the defect and the intralesional debris (Figure 2b) was removed. 

Under direct visual control, special care was taken not to injure the physis nor the subchondral plate. After removal of all suspect tissue and the perilesional membrane with an arthroscopic shaver (Figure 2c,d), the tourniquet was deflated. The entire defect was proven to be surrounded by vital and healthy bone well supplied with blood (Figure 2e,f) and the void was filled with Vitoss^®^ bone graft substitute (Stryker, Kalamazoo, MI, USA). The synthetic bone graft (Figure 2g,h) was condensed with a surgical plunger, a drainage tube was inserted into the knee, all instruments were removed and the portals were closed. A knee brace was applied for 2 weeks and partial weight bearing was recommended for 6 weeks. The cultures taken from the wound remained negative. A definitive diagnosis of osteomyelitis was made from the histopathologic examination results of the obtained tissue samples. Connective tissues with abscess and fibrotic changes and infiltration with neutrophil granulocytes were reported. In the laboratory control, the white blood cell (WBC) count was 10.2 (/nL) and CRP 19.2 (mg/L). The patient remained in the hospital for intravenous antibiotics for 12 days. WBC count and CRP at hospital discharge was 5.0 (/nL) and <0.6 (mL/L), respectively. An oral antibiotic treatment with unacid (375 mg/5 mL) was continued after discharge for another 4 weeks. 

**Figure 3:**
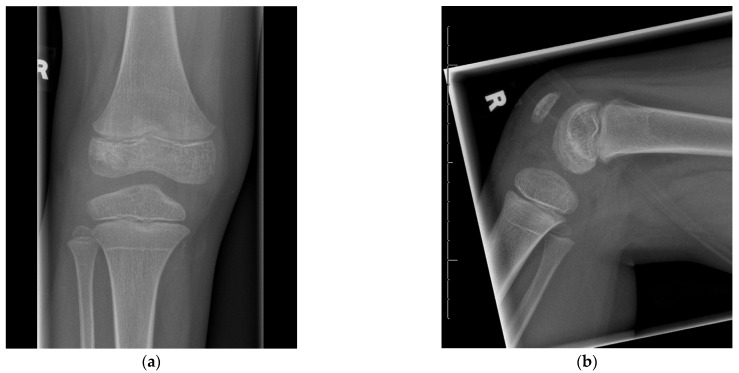
Plain radiographs of the right knee in anterior-posterior (**a**) and lateral (**b**) views of the 7-year old boy during two-year follow-up. The bone defect of the epiphysis is healed. Note the Harris-line that is present in the proximal tibia.

In the 2-year follow-up visit, full knee function was found. Neither pain and recurrent infection nor growth disturbances were observed (Figure 3a,b). 

## 3. Discussion

A 5-year old boy presented with subacute osteomyelitis characterized by an insidious onset of symptoms and was treated with antibiotic coverage and an minimally invasive surgical procedure to restore the bony anatomy of the distal femoral epiphysis. The lesion fully extended between the subchondral plate and the growth plate. Surgical access to the osseous cavity was obtained only through the articular orifice of the lesion and sampling and debridement were then performed under endoscopic guidance (“ossoscopy”), with great care not to injure the subchondral bone or the physis. 

Already, three decades ago, Green et al. reported primary subacute epiphyseal osteomyelitis in children, who demonstrated a well-deformed cavity in the epiphysis without any connection to the metaphysis [14]. 

The indication for surgery in the reported case was based on the clinical, laboratory and imaging evidence for subacute osteomyelitis with abscess formation, bone defect and septic articular involvement. Gao et al. reviewed patients with humeral epiphyseal osteomyelitis and reported a shorter length of hospital stay and intravenous antibiotic therapy [15], but a higher full recovery rate in the surgical group (83.3%), when compared to the conservative group (14.3%). A higher rate of positive culture for the pathogen has also been reported after surgical management, but the final diagnosis may also be based on histology. The fact that joint aspirate and blood cultures produced no bacterial growth is not uncommon under antibiotic treatment and has been reported by other clinicians, even if they were repeatedly obtained [16]. As a differential diagnosis to pyogenic osteomyelitis, chronic nonbacterial osteomyelitis in children has been recently characterized [17,18]. 

In 2008, Saisu et al. characterized similar difficulties in approaching the epiphysis surgically, while preserving both the physis and articular surface, fully debriding the lesion at the same time [8]. In this context, we agree that the direct visual control during the surgical procedure adds significant safety and effectiveness, because the osteomyelitis associated soft-tissue components (e.g., perilesional membrane) are not visualized with fluoroscopic techniques. The use of a 70° arthroscope or curved shavers may even assist the procedure in more complex defect configurations, but this technical modification was not necessary in the reported case. Our technique differs from previously reported techniques in two main aspects, (1) a fenestration of the physis was not performed, because the lesion was not meta-epiphyseal, but isolated to the epiphysis; and (2) the creation of bone tunnels with 4.8mm drills were avoided by using the articular orifice of the epiphyseal lesion. Furthermore, the antegrade drilling technique might interfere with the use of a tourniquet above the knee joint, which may be helpful during arthroscopy. However, avoiding any drilling is applicable only in lesions that have already developed an open articular connection. In the literature, various reports suggest that the diagnosis of subacute osteomyelitis or a delayed treatment frequently results in an articular involvement [8,9,10,14,19]. Furthermore, it has been described for subacute osteomyelitis (>2 weeks), that the epiphyseal abscess is often located in an eccentric position, potentially facilitating the penetration into the joint [6]. The eccentric position may also facilitate the surgical access. Shah et al. created a small osseous window to the epiphysis made by multiple drill holes [8]. 

Toepfer et al. applied the technique of “ossoscopy” for the minimally-invasive treatment of calcaneal cysts and bone defects; after endoscopic curettage, allogenic grafting was performed [20]. The authors concluded that the technique is simple, safe and cost-effective in benign osteolytic lesion of the calcaneus. In our case, we filled the defect with synthetic bone graft, because the lesion extended just until the subchondral plate (Figure 1d) and the cavity was filled with joint fluid. We therefore saw an indication to apply a void filler to enhance mechanical stability within the defect and potentially to accelerate osseous healing of the defect. The application of bone graft for the distal femoral epiphysis has been recommended by Sorensen et al., who grafted femoral and tibial lesions without further specification of the material [21]. 

We feel especially that the location and size of the defect should be analyzed meticulously based on MRI. A recent study from Philadelphia evaluated solitary long-bone epiphyseal lesions in children and reported a median volume of 2.8 mL in the surgically treated subgroup [22]. A recent study reported a staged treatment and final bone defect filling with bio-glass for cavitary bone defects after osteomyelitis [23]. Other authors observed uneventful osseous healing without the use of bone grafting [19,24,25] or even with nonsurgical means involving the sole use of intravenous antibiotics, while closely monitoring the osteolytic lesion under MRI [26]. The debridement of the cavity stimulated some osseous bleeding and any further stimulating measures (e.g., plasma-rich plasma) were not performed [27]. In terms of imaging limitations, we are unable to present scintigraphic imaging as presented in some classic publications [28]; however, the MRT appears to be superior in sensitivity and specificity and remains the gold-standard for evaluating the extension of solitary lesions, surgical planning and disease monitoring [3] In our case, obtaining a later MRI was impeded by the ADHD diagnosis and the legal guardian refused the follow-up MRI for evaluation of the cartilage and healing of the lesion. For this reason, we performed plain radiographs, as they are considered standard examination for follow-up [29]. 

In the available literature (Table 1), the distal femoral epiphysis has been involved in up to 75% of cases with epiphyseal infection and is therefore the most common site of bacterial osteomyelitis [14], potentially because of the larger size and its well-developed epiphyseal vascular architecture. Furthermore, higher frequency of traumatic impacts might increase the susceptibility for infections [5]. In the reported case, a high physical activity on the trampoline and one painful fall were reported prior to the development of epiphyseal osteomyelitis, but the exact etiology remains unknown. Ceroni et al. discussed whether infections of smaller epiphysis might display milder symptoms or even occur unrecognized [11]. Fungal infections or increased rates of epiphyseal osteomyelitis might be observed in children with limited host resistance [24].

In recent years, an increasing incidence rate has been reported for pediatric osteomyelitis even in high-income countries [30]. In the light of a changing epidemiology, surgical procedures with minimal morbidity to adjacent articular cartilage and growth plate are essential. The reported new technique based on arthroscopy, “ossoscopy” and bone grafting proved to be a viable treatment combination for a large bone defect of the femoral epiphysis after pediatric osteomyelitis. The planning of the surgical procedure should be based on a recent MRI and the arthroscopic modality appears to be a useful adjunct for improved visualization of the osseous lesion, while reducing the surgical morbidity and radiation exposure. The etiologic role of traumatic injuries in juvenile epiphyseal osteomyelitis and the value of minimally-invasive procedures require further scientific evaluation [5,31]. 

## Figures and Tables

**Table 1:** Previous literature focusing on bacterial osteomyelitis of the distal femoral epiphysis; Annotations; * number of cases affecting the distal femoral epiphysis (all cases reported), mo = months, F/U = follow-up; ‡ lesion not epiphyseal but meta-physio-epiphyseal, LFC = lateral femoral condyle, N/A not available.

Author(s)	Year	n (*)	Age	Knee Involvement	Therapeutic Management	F/U	Outcome
Green at al. [14]	1981	2 (8)	2–4 yrs	Lesions extended to articular cartilage without damage to cartilage itself	Curettage, sampling (6× negative), oxacillin (2× Staph. aureus)	2–8 yrs	No evidence of damage to physis or joint
Rosenbaum & Blumhagen [28]	1985	7 (9)	21 mo–9 yrs	N/A	Arthrocentesis (5) and antibiotics	N/A	N/A
Sorensen et al. [21]	1988	1 (3)	4 yrs	Joint effusion	Curettage and bone grafting, cephalexin	3 yrs	Normal
Longjohn et al. [19]	1995	1 (1)	4 yrs	Pain after initial trauma, later moderate effusion	Aspiration, nafcillin, arthrotomy, irrigation, curettage	6 yrs	Normal function, but radiographically flattening of LFC
Rasool [25]	2001	1 (2)	N/A	Lesion did not involve articular surface	Curettage, biopsy, culture, immobilization and antibiotics	2 yrs	Normal
Kao et al. [16]	2003	2	27/28 mo	Joint aspirate negative	Arthrotomy/curettage and antibiotics	6–16 mo	Normal
Abdelgawad et al. [24]	2007	1	17 mo	Mild knee effusion	Repeated fluoro- and CT-guided abscess drainage (3×), antibiotics	N/A	Normal
Saisu et al. [8]	2008	2 ‡	2–5 yrs	Chronic femoral/tibial osteomyelitis (not only epiphyseal infection)	Endoscopic surgery with drilled dual opposing insertion paths	3–4.5 yrs	Normal
Hara et al. [26]	2013	1	26 mo	Local heat and motion pain in the knee	Cefazolin (1200 mg/d), oral cefdinir (150 mg/d)	2 yrs	No recurrence
Yoo et al. [10]	2014	6 (8)	12–25 mo	Mycobacterial infections, abscess extended outside physis in 7/8 cases	Antibiotics, surgical drainage/curettage when the abscess necessitated decompression (4), or not responsive to antibiotics (4), antimycobacterial chemotherapy	4.1 yrs	Focal physeal damage in 5, 1 with growth disturbance
Shah et al. [9]	2020	12	3–14 yrs	9/18 had penetration of the joint	Open aggressive surgical treatment w/arthrotomy and antibiotics	mean 5.5 (2–11 yrs)	Joint destruction if tubercular pathogen (*n* = 4)

## Data Availability

All data are included in the manuscript, further imaging studies are filed within the digital medical record.
